# How toothed whales divide up the world: phylogeny and ecology shape life-history strategies of odontocetes

**DOI:** 10.1186/s12862-026-02530-y

**Published:** 2026-05-21

**Authors:** Steven H. Ferguson, Laura J. Feyrer, Evelien de Greef, Jeff W. Higdon

**Affiliations:** 1 Fisheries and Oceans Canada, Winnipeg, MB Canada; 2 Department of Biological Sciences, University of Manitoba, Winnipeg, MB Canada; 3 Higdon Wildlife Consulting, Winnipeg, MB Canada

**Keywords:** Life-history theory, Archetypal clustering, Phylogenetic comparative methods, Ancestral state reconstruction, Bet-hedging strategy, Sea ice, Marine conservation

## Abstract

**Background:**

Understanding the evolution of life-history strategies within a taxonomic guild offers critical insights into how species allocate energy toward growth, reproduction, and survival in response to environmental pressures. We examined 42 odontocete whale species using six key life-history traits and three major environmental variables— latitude, bathymetry, and sea ice —to identify broad patterns in ecological and evolutionary strategies.

**Results:**

While controlling for body size and phylogenetic relatedness, we used archetypal clustering analysis to identify three groups of species according to shared life-history characteristics: bet-hedging, slow, and fast. We then assessed environmental associations among these three life-history strategies. Ancestral state reconstruction was used to disentangle the roles of phylogenetic constraint and ecological adaptation in shaping these strategies. Our results reveal convergence in life-history traits among distantly related species occupying deep, offshore habitats, suggesting that environmental similarity can drive parallel evolutionary outcomes. In contrast, species inhabiting more ephemeral, high-latitude environments with seasonal sea ice exhibit lineage-specific adaptations, reflecting a stronger influence of evolutionary history.

**Conclusions:**

These findings offer a framework for tailoring conservation strategies to the distinct ecological profiles of odontocete groups, accounting for factors such as exposure to anthropogenic stressors, reliance on specialized habitats, and inherently slow recovery rates following human exploitation.

**Supplementary Information:**

The online version contains supplementary material available at 10.1186/s12862-026-02530-y.

## Introduction

Understanding the evolution of life-history strategies within a taxonomic guild provides important insights into how species allocate energy toward growth, reproduction, and survival in response to environmental pressures [106]. Among vertebrates, life-history traits such as reproductive timing and longevity are shaped by complex trade-offs that reflect both evolutionary history and ecological context [108, 130]. Investigating these traits across closely related species can reveal patterns of adaptation to environmental gradients, including factors such as temperature, resource availability, and habitat structure. In marine systems, for example, latitude, bathymetry, and sea ice extent represent critical ecological pressures that influence the evolution of reproductive strategies and survival tactics [19, 40]. By examining life-history variation in relation to these environmental drivers, we can better understand the mechanisms underpinning species resilience, ecological specialization, morphology, and vulnerability to environmental change [44]. Such insights are especially important in the face of rapid climate-driven shifts, where predicting species responses requires a deep understanding of the evolutionary and ecological foundations of life-history diversity [21, 64].

To better understand the diversity of life-history strategies and their evolutionary basis, researchers have grouped species into clusters based on shared trait combinations [23, 39, 69]. These groupings often reflect convergent solutions to similar ecological challenges, where unrelated or distantly related species exhibit comparable patterns of reproductive investment, growth rates, and survival [2, 74]. Traditional frameworks—such as r/K selection theory or the fast–slow continuum—have provided valuable starting points, yet more recent approaches incorporate multivariate and phylogenetically informed analyses to detect finer-scale patterns [7]. By clustering species based on trait residuals that account for body size and evolutionary history, it becomes possible to disentangle adaptive life-history strategies from phylogenetic inertia [38]. Such trait-based groupings often reveal associations with environmental gradients, suggesting that similar ecological pressures—such as predation risk, habitat stability, or food availability—can lead to parallel evolutionary outcomes across taxa [3, 40, 95, 97, 113]. This approach has proven especially insightful in diverse vertebrate guilds, where ecological niches are partitioned not only by morphology and behavior but also by underlying life-history strategies [30, 102, 118].

Reconstructing the evolutionary history of life-history traits provides insight into how species have adapted over time to shifting environmental conditions [56, 72]. By mapping trait evolution onto phylogenetic trees, we can identify when and how changes in reproductive timing, longevity, or body form occurred, and whether such shifts coincide with major ecological transitions [67, 72]. This phylogenetically-informed perspective allows us to distinguish between traits that are evolutionarily conserved versus those that are labile and responsive to environmental pressures. When paired with data on environmental variables—such as sea ice coverage, habitat depth, or latitudinal range—ancestral reconstruction can reveal the extent to which life-history strategies have evolved in response to specific ecological constraints [22, 93]. This approach is especially valuable for understanding adaptive radiation, niche specialization, and resilience to environmental change, as it uncovers the historical pathways through which current trait–environment relationships have emerged [1, 55]. Ultimately, linking life-history evolution to environmental drivers enhances our ability to predict how species may respond to future ecological shifts, particularly in rapidly changing ecosystems.

The cetacean fossil record extends back at least to the Middle Eocene (ca. 50 Ma), and possibly earlier [48, 99]. Odontocetes (toothed whales, Parvorder Odontoceti) are a robust model for studying life-history evolution because they span a wide range of strategies, from dolphins that live only a few decades to killer whales (*Orcinus orca*) and sperm whales (*Physeter macrocephalus*) that can live over 60 years, and vary in age at sexual maturity, interbirth intervals, and reproductive output [18, 81]. This diversity within a single clade enables comparative studies of evolutionary trade-offs under a well-resolved phylogenetic framework [49, 80, 107]. Odontocetes are characterized by complex cognition and social structures, including long juvenile dependency, cooperative care and alloparenting, cultural transmission of behaviours such as dialects and foraging techniques, and matriarchal societies [73, 124]. Although these traits are hypothesized to influence life-history evolution—particularly longevity, menopause, and kin selection [32]—they are not explicitly addressed in this study, which instead focuses on broad-scale comparative patterns in life-history traits. Ecologically, odontocetes inhabit environments ranging from shallow estuaries to deep pelagic zones and employ diverse, often overlapping feeding and foraging strategies, including suction feeding and echolocation [79, 119]. These contrasts shape energetic demands, predation risk, and resource distribution [75] and can be used to test hypotheses about how environmental pressures influence life-history traits (e.g., “fast vs. slow” life-history continua). Although odontocetes are typically considered as slow life-history or K-selected species, with low fecundity, extended parental care, high juvenile survival, and high adult longevity, these patterns vary with body size [37]. Additionally, odontocete morphological traits—including features of the skull, mandible, and vertebral column—have been associated with ecological and evolutionary variables across multiple studies [20, 52, 78, 116]. Despite substantial progress in cetacean ecology research, precise life-history data for many long-lived, wide-ranging marine mammals remain difficult to obtain due to logistical, ethical, and conservation constraints, resulting in uneven species coverage where some taxa and populations are well studied while others remain poorly known [60, 70]. In summary, their trait diversity, social complexity, ecological breadth, and well-resolved phylogeny make odontocetes a valuable group for examining life-history evolution.

To investigate the evolutionary and ecological drivers of life-history diversity in odontocetes, we integrated phylogenetic, life-history, and environmental data across 42 species. Our first objective was to identify life-history strategies across odontocete taxa through life-history traits. We followed Winemiller and Rose [126] in estimating ‘archetypes’ that represent an extreme combination of life-history characteristics, and we refer to these archetypes as ‘life-history strategies’ (LHS hereafter). In the second objective, we explored the association between the resulting LHS and environmental conditions to evaluate whether specific habitats may have influenced the evolution of these life-history strategies. Our last objective was to explore the evolutionary history of major environmental variables across the odontocete phylogeny using ancestral state reconstruction. We discuss our results in terms of how distinct life-history strategies in odontocetes have evolved in response to environmental conditions, providing a framework for assessing species’ vulnerabilities in the context of ongoing climate-driven change.

## Methods

### Dataset compilation

Life-history trait data for cetaceans were compiled from multiple sources to maximize species coverage while maintaining data quality. The initial dataset [37, 39] included both mysticete and odontocete species and was filtered to exclude species with three or more missing life-history variables. This resulted in a dataset of 54 species with sufficient trait information for analysis.

For the present study, only odontocete species were retained [37]. To expand taxonomic representation, additional life-history data were obtained from the AnAge Database of Animal Ageing and Longevity [110], part of the Human Ageing Genomic Resources (HAGR). Variables extracted included longevity, female age at sexual maturity, gestation length, interbirth interval, and neonate body length. The AnAge database contributed data for 45 species, including several river dolphin taxa.

Further supplementation was conducted using the PanTHERIA database [66], which provides comparable life-history traits and additional information on body length at birth. While PanTHERIA includes a large number of cetacean species, data completeness varied, and only records with usable trait information were retained.

Adult body mass and length were required to meet a minimum sample size threshold (≥ 5 individuals) because these variables were used to generate size-adjusted residuals for all life-history traits. For other traits, sample size information was not consistently available across data sources; therefore, values were compiled from multiple published databases and cross-checked for consistency, with obvious outliers excluded where necessary. Adult body length data were compiled from published sources that varied in reporting detail, including length at physical or sexual maturity (sometimes sex-specific) or ranges of adult size. Data were more limited for physical maturity (males: *n* = 14; females: *n* = 16) than for sexual maturity (males: *n* = 41; females: *n* = 42). To maximize species coverage, we used the upper bound of the reported *typical* adult length range (excluding maximum recorded individuals) as a standardized proxy for adult body size. This measure, typically reported for both sexes combined, increased sample size to *n* = 42 species. Although sexual size dimorphism occurs in some cetaceans, consistent sex-specific data were unavailable; combined-sex values were therefore used to ensure comparability.

The use of maximum typical adult size as a proxy for body size is common in comparative analyses when standardized maturity metrics are unavailable, as it captures asymptotic size while minimizing bias from extreme individuals (e.g., [15, 96]). We evaluated this proxy by correlating it with available maturity-based length estimates. Maximum typical adult length was strongly correlated with length at sexual maturity in males (*r* = 0.987, *n* = 48) and females (*r* = 0.971, *n* = 49), as well as with other maturity metrics (all *r* = 0.797–0.992, *p* < 0.01), supporting its use in comparative analyses.

Consistency among data sources was evaluated for each trait. In cases of conflicting values, records were verified and updated using primary literature, with priority given to the Encyclopedia of Marine Mammals [127]. For species lacking reliable estimates, a value was mean-centered and missing values set to zero following standard practice for retaining incomplete cases to prevent undue influence on model outputs while preserving sample size [128]. When trait values were reported as ranges or multiple estimates across sources, we calculated the mean value and rounded to the nearest whole number to obtain a single representative estimate for each species. For species exhibiting intra-specific variation (e.g., inshore versus offshore forms) or distinct ecotypes (e.g., resident and transient forms), trait values were compiled at the species level due to limited and inconsistent availability of population-specific estimates across datasets. Examples of life-history trait value changes from Ferguson et al., [37] included *Phocoena dioptrica* (spectacled porpoise), *Phocoena spinipinis* (Burmeister’s porpoise), and *Hyperoodon ampullatus* (northern bottlenose whales) [42, 127].

Our nomenclature adheres to the guidelines by the Society of Marine Mammalogy’s Committee on Taxonomy (*List of Marine Mammal Species and Subspecies - Society for Marine Mammalogy*). In total, we obtained data on species’ traits from 6 families, 24 genera, and 42 species of odontocete cetaceans. We adapted the phylogeny of McGowen et al., [80] for the 42 odontocete species to control for phylogenetic effects.

We selected neonate length (cm), age of female sexual maturity (y), gestation length (d), interbirth interval (y), longevity (y), and morphology (body shape) as the life-history variables. To normalize the distribution of life-history data, which exhibited a strong right skew, all six variables were log-transformed, a standard practice in comparative approaches [62]. Normality of all log_10_-transformed data distributions was confirmed using Wilk-Shapiro normality tests. Body shape, a novel morphology trait, was calculated as the residual from a log–log regression of body mass versus body length [37].

Environmental variables including absolute latitude, bathymetry, and sea ice concentration, were averaged across a global database at 1° spatial resolution (180 × 360 grid), with values assigned to the centroid of each grid cell within the geographic range for each odontocete species based on range maps (shapefiles) provided by the IUCN Red List (Spatial Data version 1.20 IUCN Red List of Threatened Species). Bathymetric data and a land-sea mask were obtained from NOAA’s World Ocean Atlas 2013 [12]. Data for length of the sea ice season (in months) came from the Sea Ice Index from the National Snow and Ice Data Center (median location, [41]). The Sea Ice Index defines regions covered by ice as areas with greater than 15% ice concentration (standard definition for ice extent; [92]). From this, we extracted the length of the sea ice season in months from the Ice Edge Index climatology (Data Tools | National Snow and Ice Data Center). All spatial data were standardized to a 1-degree grid (*n* = 64,800 cells including land) and referenced to cell centroids in the vector lattice. Due to the structure of the compiled dataset, we used representative (central tendency) values rather than full ranges for latitude and bathymetry.

### Phylogenetic signal

Next, we evaluated the strength of phylogenetic signal in the traits to determine whether phylogenetically controlled methods were necessary. Phylogenetic signal refers to the pattern where closely related species exhibit more similar trait values than more distantly related species (i.e., violates the statistical assumption of independence due to phylogenetic structure). We tested whether phylogenetic signal was present in odontocete life-history and environmental traits following the methods of Pagel and Harvey [90] using Pagel’s λ [89] and Blomberg’s K [9], implemented using the phytools [101] package in R.

### Life-history strategies

We then assessed the clustering of odontocete species based on their life-history traits while controlling for both body size and phylogeny. To control for body-size effects, we calculated residuals for the life-history traits and then scaled and centered them prior to analysis. To control for phylogeny, we conducted an archetype hierarchical cluster analysis using the phylosem and archetypes R package [34, 112]. We tested models with k = two to five LHS and selected the optimal solution (k = 3) based on residual sum of squares (RSS) and model convergence criteria (Fig. S1). Each species was assigned to the nearest LHS based on posterior probabilities. LHS were later interpreted and labeled based on dominant trait patterns. We extracted trait parameter values for each archetype and evaluated the relative expression of each life-history trait across clusters. To further understand the phylogenetic relationship among LHS, we plotted odontocete families along their 2-dimensional clustering.

### Life-history features associated with LHS

In comparative studies, the strength of phylogenetic signal varies among traits and datasets, and formal tests such as Pagel’s *λ* or Blomberg’s *K* are used to assess whether phylogenetic structure is statistically detectable in the data (e.g., [9]). When phylogenetic signal is weak or not significant for particular traits, the benefits of phylogenetic correction may be limited, and non-phylogenetic analyses (i.e., standard regression models) can be equally informative as special cases of a star phylogeny (i.e., no covariance among species) in comparative contexts. In such cases, presenting both phylogenetically controlled (e.g., PGLS) and non-phylogenetic results allows readers to evaluate the influence of phylogeny on effect estimates and model interpretation without imposing a correction that is not supported uniformly across variables ([9]; Revell et al., 2008). Here, we assessed the relationship between the six key life-history traits and the three LHS using phylogenetic and non-phylogenetic analyses. We used the residual values for each life-history trait described earlier to continue accounting for overall body size. We used phylogenetic generalized least squares (PGLS) models [10] implemented via the nlme package in R [98] that incorporated a published odontocete phylogeny (pruned to our 42 species) and assumed a Brownian motion model of trait evolution [37]. The lambda parameter (λ) was estimated by maximum likelihood and used to transform branch lengths accordingly.

Trait residuals for body mass, gestation length, longevity, neonate length, age of sexual maturity, and interbirth interval on adult body length were used in non-phylogenetic ANOVA comparisons of LHS. We tested for significant differences in residual life-history traits among the three LHS using one-way ANOVAs, followed by Tukey’s HSD post hoc tests for pairwise comparisons. Although residuals were derived from phylogenetically informed models, these ANOVAs were non-phylogenetic and served to highlight trait-level group differences. In addition, we examined trait scaling relationships by testing whether the slope or intercept of each trait’s relationship with body length differed significantly among LHS (α ≤ 0.05).

Residual trait values for all species were explored using biplots and barplots grouped by LHS. Trait covariation across LHS was further examined using scatterplots with family-level ellipses representing 95% confidence intervals to visualize patterns of group separation. Family-level grouping provides a biologically meaningful framework, reflecting shared evolutionary history and ecological similarity, which aids interpretation of trait patterns. Ellipses were only plotted for families with sufficient species to reliably estimate the confidence interval; families with very few species did not generate ellipses, which accounts for their absence in some plots. Because patterns of covariation were consistent across trait combinations, and to maintain clarity, we present a single representative example illustrating the relationship between gestation length and neonate length. All analyses were conducted in R version 4.5.1, and visualization was performed using the ggplot2 package ([125]).

### Relationships between LHS and environment

We applied phylogenetic and non-phylogenetic models to test for significant differences in environmental characteristics among the three identified LHS to infer the likely evolutionary pathways linking environmental conditions to life-history strategies. We ran a PGLS model [10] with a logistic link to test whether the environmental variables – latitude, bathymetry, and sea ice season length (hereafter referred to as sea ice) – differed among the three LHS, accounting for body size and phylogenetic relatedness. This mixed-effects modeling approach used a Brownian correlation structure to control for phylogeny and was able to determine the relationship between life-history strategies and major environmental variables [51].

### Ancestral state reconstruction

Lastly, we used ancestral state reconstruction to explore the evolutionary history of the three major environmental variables—latitude, bathymetry, and sea ice—across the odontocete phylogeny. Ancestral reconstruction is a statistical method that uses phylogenetic inference to construct an evolutionary tree [67]. Here, we used phylogenetic inference to construct a phylogeny that represented the evolutionary relationships among odontocete species and to understand the evolutionary history of the three groups of whales as well as their use of habitat.

Ancestral reconstruction was performed using the Ancestral Character Estimation (ace) method in the ape R package [91], which estimated the characteristics of ancestral species based on the characteristics of their descendants, while also accounting for uncertainty [101]. Maximum likelihood values at a given node were computed using only the information from the tips and branches descending from that node, projecting the phylogenetic tree in a space defined by phenotype (on the y-axis) and time (on the x-axis) [65].

## Results

### Dataset compilation

The odontocete dataset covered a wide range of species sizes and ages. For example, body mass in our dataset ranged from 32.5 kg (Indo-Pacific finless porpoise, *Neophocaena phocaenoides*) to 14,025 kg (sperm whale), and lifespans ranged from 8 years (spectacled porpoise, *Phocoena dioptrica*) to 90 years (killer whale, *Orcinus orca*). Bathymetry data (average within species geographic range) went from 5 m for Tucuxi (*Sotalia fluviatilis*) living in the Amazon basin up to 5060 m in depth for the Hubb’s Beaked whale (*Mesoplodon carlhubbsi*) with a mean of 3232 m across species. Given the wide distribution and representation of the odontocete taxa, absolute latitude spanned from 0 to 77.8^o^ (narwhal, *Monodon monoceros*), covering areas from tropical to polar habitats. Sea ice was present for at least one month in range distributions for 9 species.

### Phylogenetic signal

Tests for phylogenetic signal in log-transformed life-history residual traits removing the effects of body mass revealed no strong or consistent evidence of phylogenetic patterning across traits (Table 1). Both Pagel’s lambda (λ) and Blomberg’s K values were generally low and only two of the traits showed statistically significant phylogenetic signal using Blomberg’s K: age of sexual maturity and gestation length. Both age of sexual maturity and longevity came close with Pagel’s λ values (*P* < 0.10). Traits such as body length and interbirth interval had particularly low λ values, supporting the absence of strong phylogenetic structuring in these life-history traits. Overall, these findings support the emergence of life-history diversity among odontocetes as a product of ecological and reproductive trade-offs, rather than evolutionary constraint.

**Table 1:** Summary of tests of phylogenetic signal in odontocete cetacean life-history traits (log-transformed residuals removing effects of body size) and environmental features. Pagel’s lambda (λ) and Blomberg’s K indicates the estimated strength of phylogenetic signal in model residuals

Response Variable	λ	*p*-value λ	Blomberg’s K	*p*-value K
Adult body length (cm)	0.30	0.29	0.22	0.21
Age of sexual maturity (y)	0.28	0.069	0.28	0.049
Longevity (y)	0.33	0.073	0.23	0.16
Gestation length (d)	0.00	1.00	0.27	0.049
Interbirth interval (d)	0.00	1.00	0.22	0.22
Neonate length (cm)	0.08	0.69	0.23	0.12
Latitude	0.08	< 0.01	0.25	0.047
Bathymetry	0.00	1.00	0.12	0.91
Sea ice	1.04	< 0.01	0.39	0.098

Phylogenetic signal in environmental variables associated with odontocete cetaceans was generally strong (Table 1). Pagel’s λ values were significant for latitude (*P* < 0.01) and sea ice (*P* < 0.01), indicating strong phylogenetic structuring. For Blomberg’s K, latitude was significant (*P* = 0.047), whereas sea ice was moderate (*P* < 0.10), suggesting phylogenetic patterning. Bathymetry had a non-significant relationship for both λ (0.00) and Blomberg’s K (0.12), indicating little to no phylogenetic signal (*P* > 0.9). For latitude and sea ice environmental predictors, results suggest that ecological niche use was strongly constrained by phylogeny. In downstream analyses for both life-history traits and environmental variables, we examined statistical models with and without these phylogenetic controls.

### Life-history strategies

**Fig. 1:**
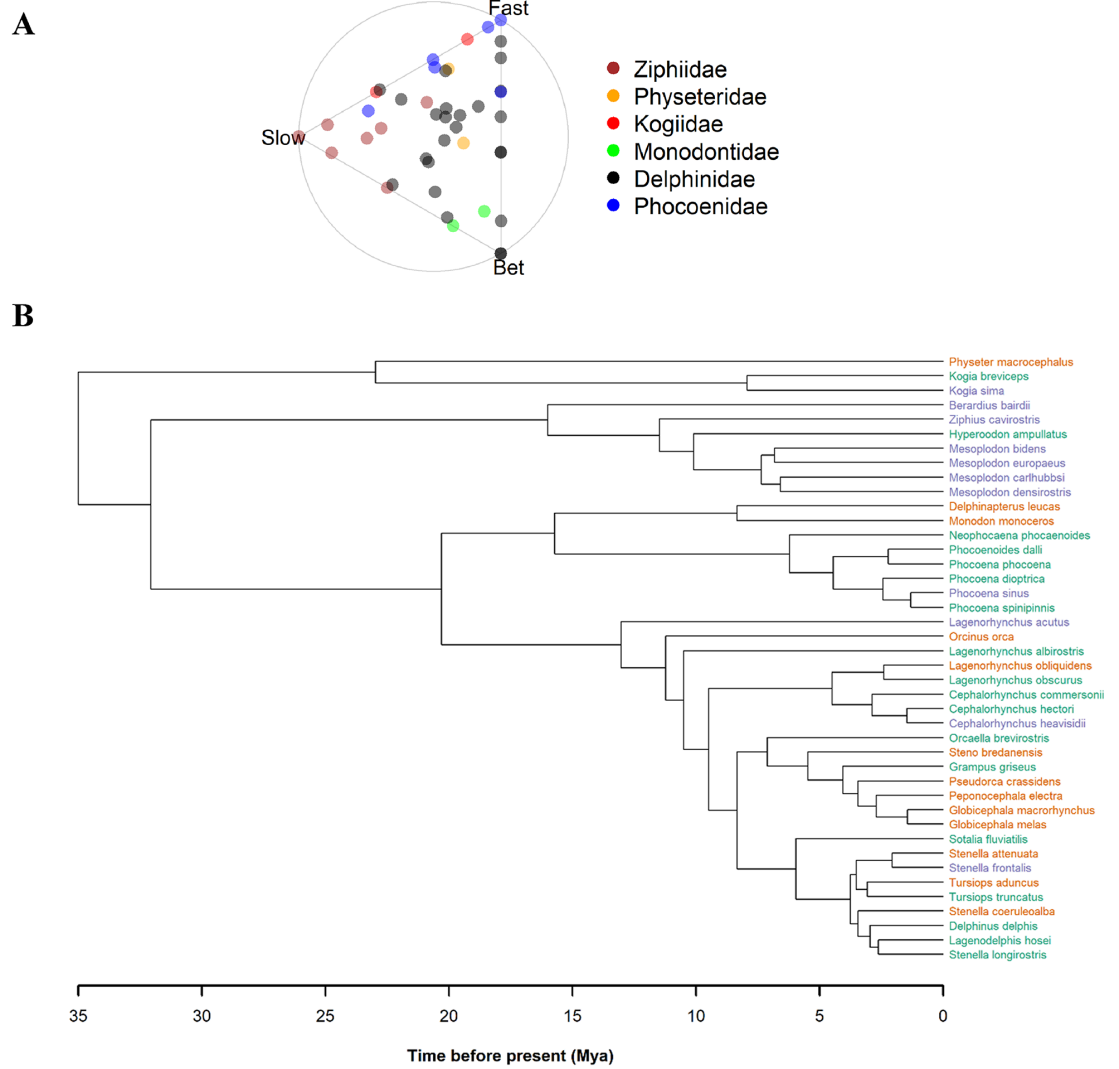
Archetype analysis of 42 odontocete species based on multivariate life-history traits, illustrating a continuum of life-history strategies (LHS) and their distribution across families. **(A)** Triangular archetype plot in which each point represents a species, color-coded by family and positioned according to its relative affinity to three LHS: bet-hedging (bottom right), fast (top right), and slow (left). Species located near the vertices show strong alignment with a given strategy, whereas those near the center exhibit weaker (i.e., “fuzzy”) associations. **(B)** Phylogenetic tree with tip labels color-coded by assigned LHS. Species are grouped into three clusters—bet-hedging (orange), fast (green), and slow (purple)—based on multivariate life-history traits corrected for body size and phylogeny. The tree highlights the evolutionary distribution of LHS across odontocetes

The archetype analysis identified three LHS among 42 odontocete whale species after controlling for body size (through life-history trait residuals) and phylogenetic history. The three LHS differed across six key residual life-history traits (morphology, gestation length, interbirth interval, neonate length, age at sexual maturity, and longevity), suggesting divergent ecological and reproductive strategies. These strategies, which can be visualized on a ternary (triangle) plot based on species’ relative affinities to the three LHS, reflect adaptive divergence across major taxonomic groups (Fig. 1A). Species positioned near the centre of the triangular life-history space show weaker affinity to any single strategy, indicating either intermediate trait combinations or greater uncertainty in underlying data; for example, the Northern bottlenose whale plots away from the vertices, suggesting it does not strongly conform to a single life-history category. The first life-history strategy, positioned at one vertex of the triangle, was dominated by the family Monodontidae (e.g., beluga (*Delphinapterus leucas*) and narwhal), which we describe as “Bet-Hedging” (Fig. 1B), characterized by traits reflecting conservative reproductive investment, such as later age at sexual maturity and longer interbirth intervals. The vertex representing the “Fast” whales centered around species from the Delphinidae and Phocoenidae families, defined by traits associated with faster life histories—shorter gestation periods, earlier sexual maturity, and shorter interbirth intervals. Lastly, we labeled the third life-history strategy as “Slow” (Fig. 1B). These whale species exhibit traits consistent with a slow, deep-diving life history, characterized by intermediate longevity and morphology, but relatively low reproductive output.

### Life-history features associated with LHS

**Fig. 2:**
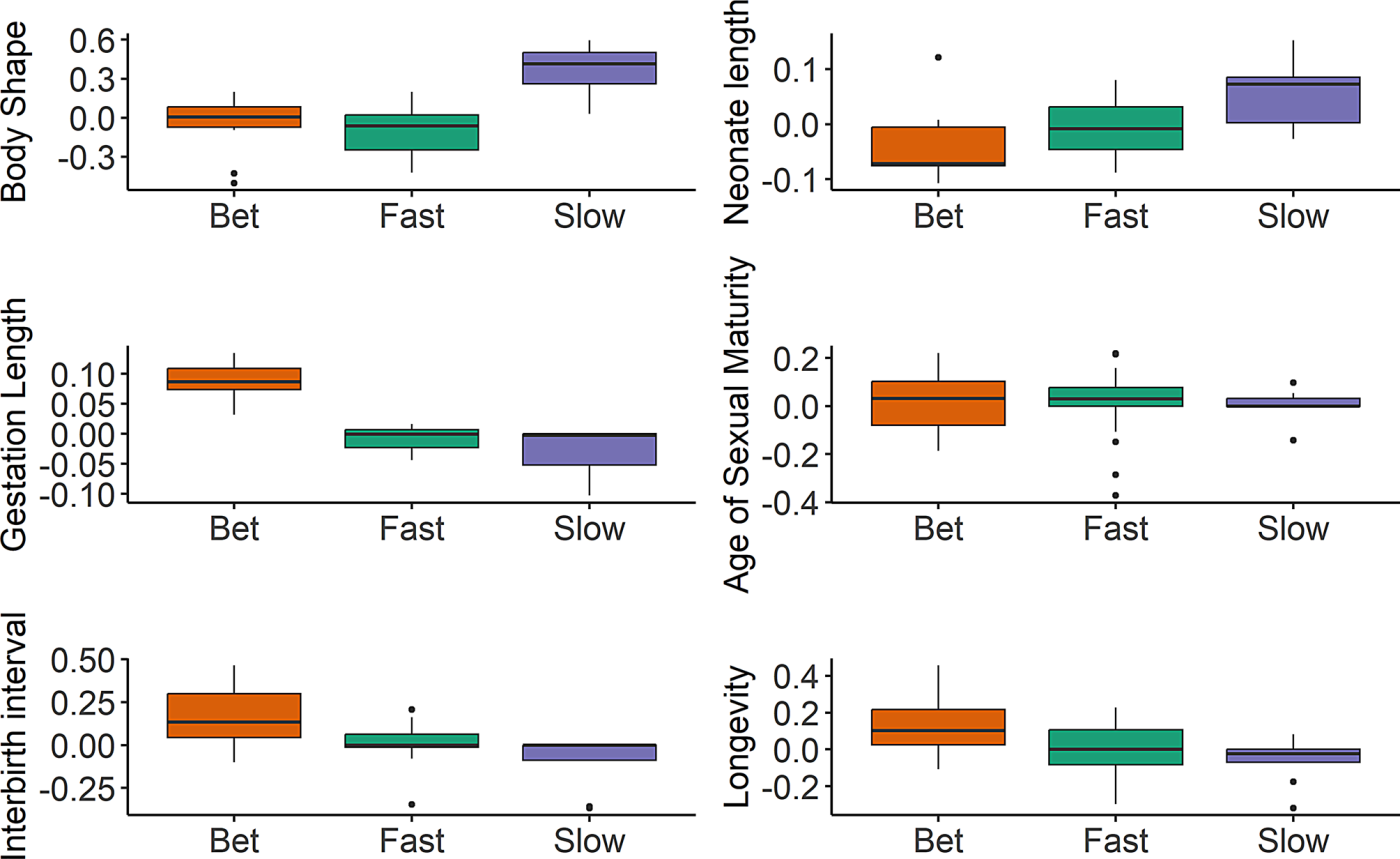
Boxplots showing differences in standardized residuals for six life-history traits among the three identified LHS of odontocetes: Bet-hedgers, Fast, and Slow. The traits include morphology (body shape), neonate length, gestation length, age of sexual maturity, interbirth interval, and longevity. LHS reflect distinct life-history strategies with varying degrees of reproductive investment and ecological specialization. Significant differences among LHS were identified for multiple traits (see Table 2 for phylogenetically and non-phylogenetically controlled statistical results)

Residual variation in the phylogenetically controlled analysis across life-history traits illustrated clear differences among the three odontocete LHS (Bet-hedgers, Fast, and Slow whales) relative to species averages (Table 2A, Fig. 2). A permutation test assessing overall multivariate trait divergence among LHS confirmed significant separation in life-history profiles (F_2,39_ = 8.96, *p* = 0.001). The unique combinations of residual values in each LHS supports their ecological and evolutionary distinctiveness.

The Bet-Hedging LHS (*n* = 16 species) was characterized by an average body shape (i.e., neither streamlined nor robust). These species also exhibited a slightly longer gestation period, longer lifespan, and delayed age of sexual maturity, along with smaller neonates and long interbirth interval. These traits reflect a high per-offspring investment and reproductive spacing, indicative of a strategy which reduces reproductive risk by spreading offspring production across time.

The Fast LHS (*n* = 11) displayed a contrasting life-history profile. They had a more streamlined body shape (negative residuals), shorter gestation and lifespan, earlier sexual maturity, and shorter interbirth interval. Neonate size was close to predicted values. These traits are consistent with a fast-paced, more r-selected strategy, emphasizing early maturation, high reproductive frequency, and reduced per-offspring investment.

The Slow LHS (*n* = 15) exhibited a distinctive life-history pattern. These species had a robust body shape (i.e., individuals were heavier than expected for their length, positive body shape residual), moderate gestation duration, intermediate to long lifespan, larger neonates, slightly delayed sexual maturity, and average interbirth interval. While not fitting clearly within r/K-selection theory, their traits suggest a specialized strategy, likely shaped by the demands of deep-diving and extreme environments.

**Fig. 3:**
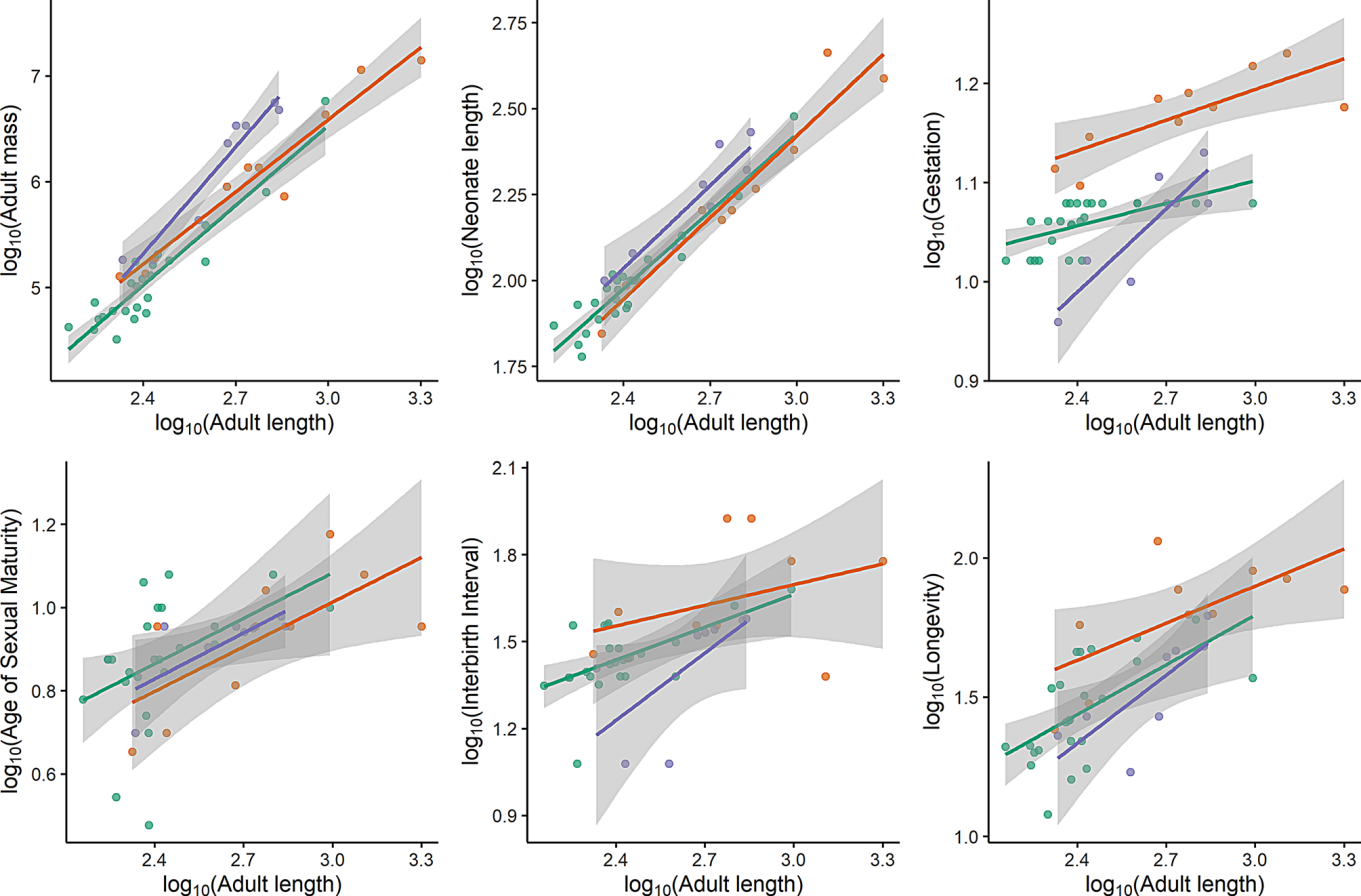
Log-log regressions of life-history traits as a function of adult body length (log₁₀-transformed) for three odontocete life-history strategies (LHS): Bet-hedgers (orange), Fast (green), and Slow whales (purple). Each panel shows a different trait: (top row) body mass, neonate length, gestation length; (bottom row) age at sexual maturity, interbirth interval, and longevity. Solid lines represent group-specific linear regression fits with shaded 95% confidence intervals. Slow whales consistently deviate in slope or intercept for several traits (e.g., body mass, neonate length), indicating a distinct life-history strategy characterized by greater maternal investment and altered reproductive pacing. ANOVA and post hoc tests confirm significant group differences for morphology, neonate length, gestation length, and interbirth interval (Table 2)

Non-phylogenetic ANOVAs also revealed several significant trait differences among LHS (Table 2B). Morphology differed strongly among LHS (ANOVA: F₂,₃₉ = 16.23, *p* < 0.001), with Slow LHS whales having significantly greater mass for their length than both Bet-hedgers and Fast LHS (Tukey tests, both *p* < 0.01). Neonate size varied significantly (F₂,₃₉ = 6.47, *p* < 0.01), with Slow whales producing larger neonates relative to maternal size (Slow–Fast, *p* = 0.02; Slow–Bet-hedgers, *p* < 0.01), suggesting increased maternal investment. Gestation length showed the most pronounced differences (F₂,₃₉ = 52.0, *p* < 0.001), with Fast whales having significantly shorter gestation than both Bet-hedgers and Slow whales (Bet-hedgers–Fast and Slow–Bet-hedgers, both *p* < 0.01). Interbirth interval also differed significantly (F₂,₃₉ = 9.17, *p* < 0.001), with Bet-hedgers showing longer intervals than Fast LHS (*p* < 0.01), and Slow whales differing from Bet-hedgers (*p* < 0.01), reflecting contrasting reproductive pacing. In contrast to the phylogenetically controlled results, age at sexual maturity did not differ significantly among groups (F₂,₃₉ = 0.03, ns), suggesting this trait may be constrained across odontocetes (Fig. 3). Longevity exhibited a marginal group-level effect (F₂,₃₉ = 3.44, *p* = 0.042), though pairwise comparisons did not reach significance (Tukey *p* ≈ 0.06–0.07), hinting at potential trends warranting further investigation.

**Table 2:** Three distinct life history strategies (LHS: Bet-hedgers, Fast, and Slow) among odontocete whales based on residuals of key life-history traits. (**A**) Phylogenetic controlled results using PGLS assessed by Goodness-of-Fit (RSS = 0.142), Interactions = 3, Alpha = 1.828, Beta – 1.273, Z_as_ = 66.56, Z_s_ = 77.42. Trait values are standardized residuals. (**B**) Summary of non-phylogenetic ANOVA and Tukey Post Hoc results comparing LHS across residual traits controlling for body size. Phylogenetic controlled results:

(A) Phylogenetic controlled results:			
Trait	Bet-Hedgers (*n* = 16)	Fast Whales (*n* = 11)	Slow Whales (*n* = 15)
Morphology	Average (− 0.02)	Streamlined (− 0.35)	Robust (+ 0.31)
Gestation Length	Slightly longer (+ 0.06)	Short (− 0.05)	Moderate (+ 0.01)
Longevity	Long-lived (+ 0.14)	Short-lived (− 0.30)	Moderate (+ 0.06)
Neonate Length	Small (− 0.08)	Average (− 0.00)	Large (+ 0.10)
Age at Sexual Maturity	Later (+ 0.08)	Early (− 0.18)	Slightly late (+ 0.07)
Interbirth Interval	Long (+ 0.24)	Short (− 0.20)	Average (− 0.01)

(B) Non-phylogenetic results:					
Residual Variable	ANOVA F (df = 2, 39)		ANOVA *p*-value	Significant Tukey Contrasts (*p* < 0.05)	Notes
Morphology	16.23	< 0.01		Slow–Fast (*p* < 0.01), Slow–Bet-hedgers (*p* < 0.01)	Strong differences driven by Slow whales
Longevity	3.44	0.042		None (trends: Bet-hedgers–Fast and Slow–Bet-hedgers *p* ~ 0.06–0.07)	Weak group effect; marginal pairwise trends
Age of Sexual Maturity	0.03	ns		None	No group differences detected
Neonate length	6.47	< 0.01		Slow–Fast (*p* = 0.02), Slow–Bet-hedgers (*p* < 0.01)	Slow whales differ significantly from others
Interbirth interval	9.17	< 0.01		Bet-hedgers–Fast (*p* < 0.01), Slow–Bet-hedgers (*p* < 0.01)	Bet-hedgers and Slow whales show opposing patterns vs. each other and vs. Fast
Gestation length	52.0	< 0.01		Bet-hedgers–Fast (*p* < 0.01), Slow–Bet-hedgers (*p* < 0.01)	Bet-hedgers and Slow whales show opposing patterns

Regressions of life-history traits as a function of adult body length for three odontocete LHS indicated grade shifts as similar slopes with differing intercepts (Fig. 3). Slow whales consistently deviate in slope or intercept for several traits (e.g., more robust body shape, greater neonate size), indicating a distinct life-history strategy characterized by greater maternal investment and altered reproductive pacing. Fast whales were intermediate in many life-history features. Bet-hedgers had longer gestation length relative to their body size but smaller neonates indicating reduced offspring investment. Bet-hedgers also had delayed sexual maturity, longer interbirth interval, and longer lifespan indicating greater adult somatic investment.

**Fig. 4:**
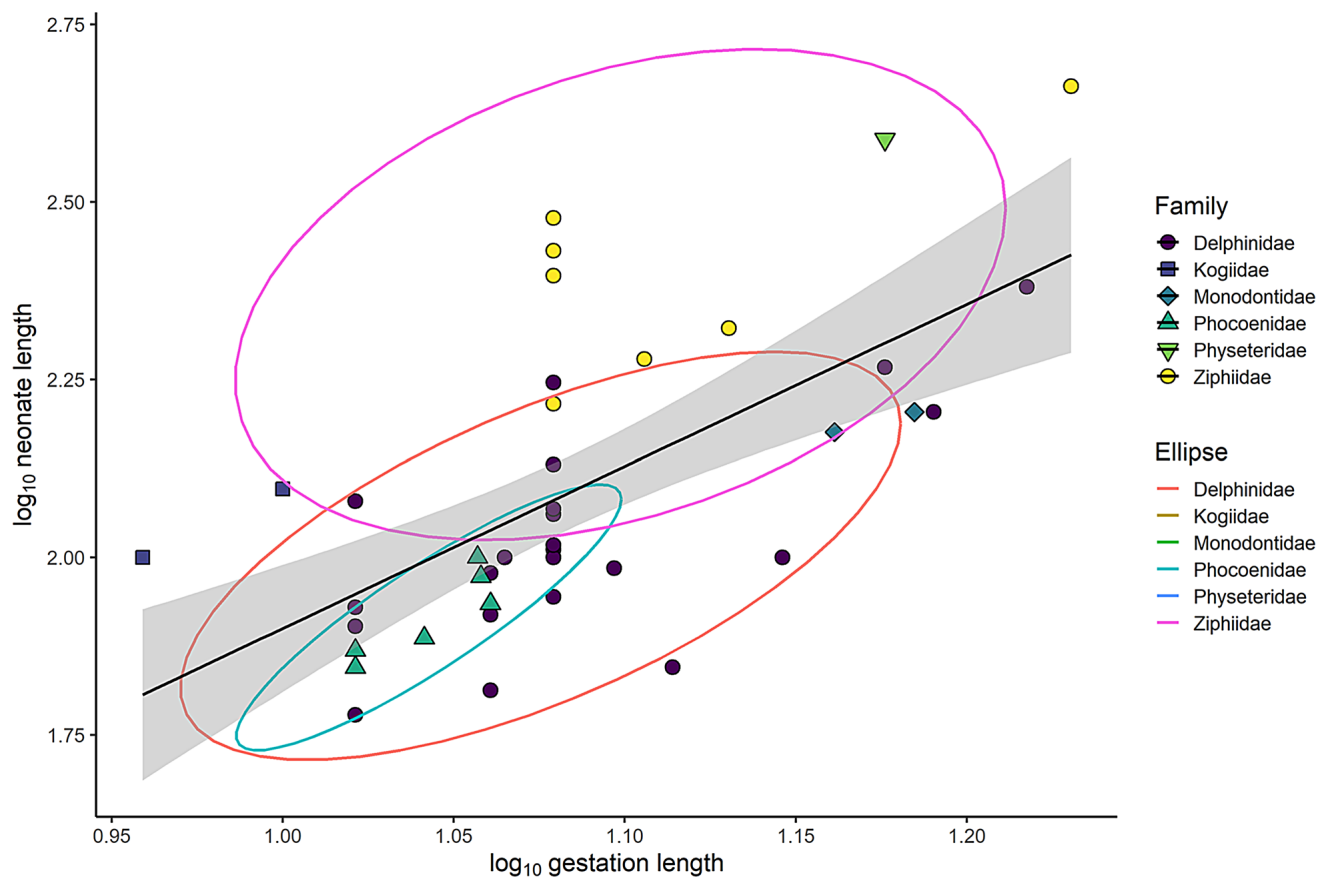
Relationship between gestation length and neonate length among 42 odontocete species. The plot shows log-transformed neonate length (y-axis) versus log-transformed gestation length (x-axis), with each point representing a species and color-coded by odontocete family. The solid regression line with shaded 95% confidence interval indicates a significant positive correlation, where species with longer gestation periods tend to produce larger neonates. Family-specific 95% data ellipses for families with greater than 2 species illustrate variation in this relationship

Additionally, family-level patterns were explored using ellipses plotted for gestation length versus neonate length (Fig. 4). Slow whales (e.g., Ziphiidae) clustered above the regression line, indicating relatively large neonates for their gestation duration, while Fast whales (Delphinidae and Phocoenidae) fell below the line—reflecting smaller neonates despite comparable gestation times. These visual trends reinforce the differentiation of life-history strategies revealed through archetype clustering.

### Relationships between LHS and environment

The PGLS model tested whether the environmental variables (latitude, bathymetry, sea ice) differed among the three LHS (Table 3). For latitude, the intercept corresponded to the mean absolute latitude for Bet-hedgers, estimated at 26^o^, significantly different from zero (*p* = 0.008). Fast whales were estimated to occur at 25^o^ and Slow whales at 21^o^ absolute latitude, but this difference was not statistically significant (*p* = 0.55). The strong phylogenetic signal (λ = 0.08) described earlier suggested phylogenetic autocorrelation in latitude distribution, i.e., species’ latitudinal distribution was strongly structured by phylogeny here. However, residual standard error was large (~ 25), indicating substantial variability in latitude among species groups. Overall, the three LHS did not differ significantly in their absolute latitude distributions.

When testing differences between LHS with bathymetry, the PGLS model showed that Bet-hedgers may use deeper waters than Fast whales; but this was marginally significant (*p* = 0.089). The bathymetry used by Slow whales was not different from Bet-hedgers (*p* = 0.716). Fast whales may use shallower waters than the other two LHS, but the evidence was weak. The λ estimate for bathymetry suggested little or no phylogenetic signal, indicating that species’ bathymetry values aren’t strongly structured by shared ancestry.

For sea ice season length, Fast whales experienced an estimated 0.3 months/year lower than Bet-hedgers (2.2 months/year), but this difference was not statistically significant (*p* = 0.16). Slow whales had an estimated sea ice of 0.1 months/year lower than Bet-hedgers, with a marginal p-value of 0.065, suggesting it might use less sea ice habitat compared to Bet-hedgers. The 1.04 λ suggests a strong phylogenetic signal in sea ice habitat use (i.e., closely related species tend to have similar sea ice habitat preferences), however the residual standard error here was quite large (2.34), which might indicate variability in data or scale issues. While these results suggest Slow whales tend to use less sea ice habitat than Bet-hedgers, this difference was only marginally significant; whereas Fast whales show a moderate but non-significant trend toward less sea ice use.

**Table 3:** Summary of phylogenetic generalized least squares (PGLS) models testing whether species grouped by life-history strategy differ in bathymetry (depth), sea ice season length (mean season), and latitude (absolute value). All estimates are relative to Bet-hedger LHS

Response Variable	Life-history Strategy	Estimate	Std. Error	t-value	*p*-value	AIC
Latitude	Fast whales	+ 5.237	8.711	0.60	0.551	377.2
	Slow whales	+ 3.326	9.940	0.33	0.740	
Bathymetry	Fast whales	-0.365	0.210	-1.74	0.089	83.5
	Slow whales	-0.085	0.231	-0.37	0.716	
Sea Ice	Fast whales	-0.533	0.372	-1.43	0.159	163.7
	Slow whales	-0.913	0.481	-1.90	0.065	

**Fig. 5:**
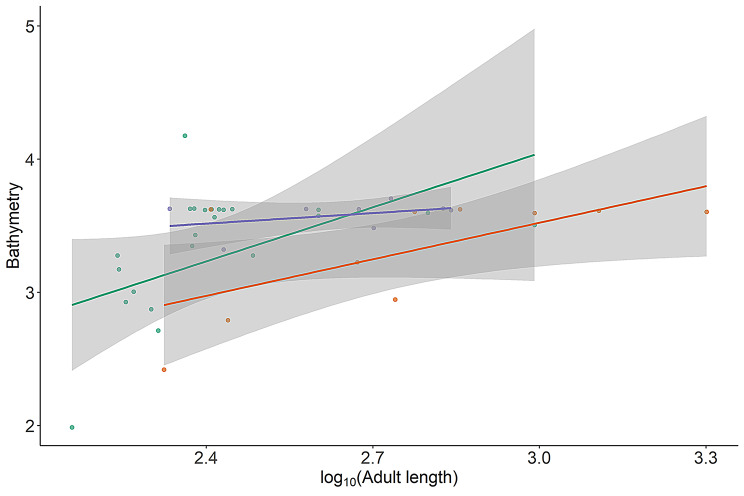
Relationship between adult body length (log₁₀-transformed) and bathymetry (log₁₀-transformed) for three LHS of odontocetes: Bet-hedgers (orange), Fast (green), and Slow whales (purple). Shaded regions represent 95% confidence intervals around group-specific regression lines. Slow whales are consistently associated with deeper waters, but show no significant relationship between body size and depth. In contrast, both Fast and Bet-hedgers generally inhabit shallower waters, with larger-bodied species occurring in deeper habitats, indicating a positive association between body size and bathymetry in these groups

Considering non-phylogenetic results, no strong relationship between body size and use of sea ice or latitude was found. We did find a graphic relationship between adult body length (log₁₀-transformed) and bathymetry (depth) for the three LHS of odontocetes (Fig. 5). Slow whales were consistently associated with deeper waters across their size range (mean = 3910 m), with no clear relationship between body length and bathymetry. In contrast, both Fast and Bet-hedgers tended to inhabit shallower waters overall (means 3190 and 2792 m, respectively), but exhibited a positive relationship between body size and depth—larger species are found in deeper habitats. This suggests differing ecological strategies, with Slow whales occupying deep habitats regardless of size, while the other two LHS show more flexible depth usage based on body size.

### Ancestral state reconstruction

**Fig. 6:**
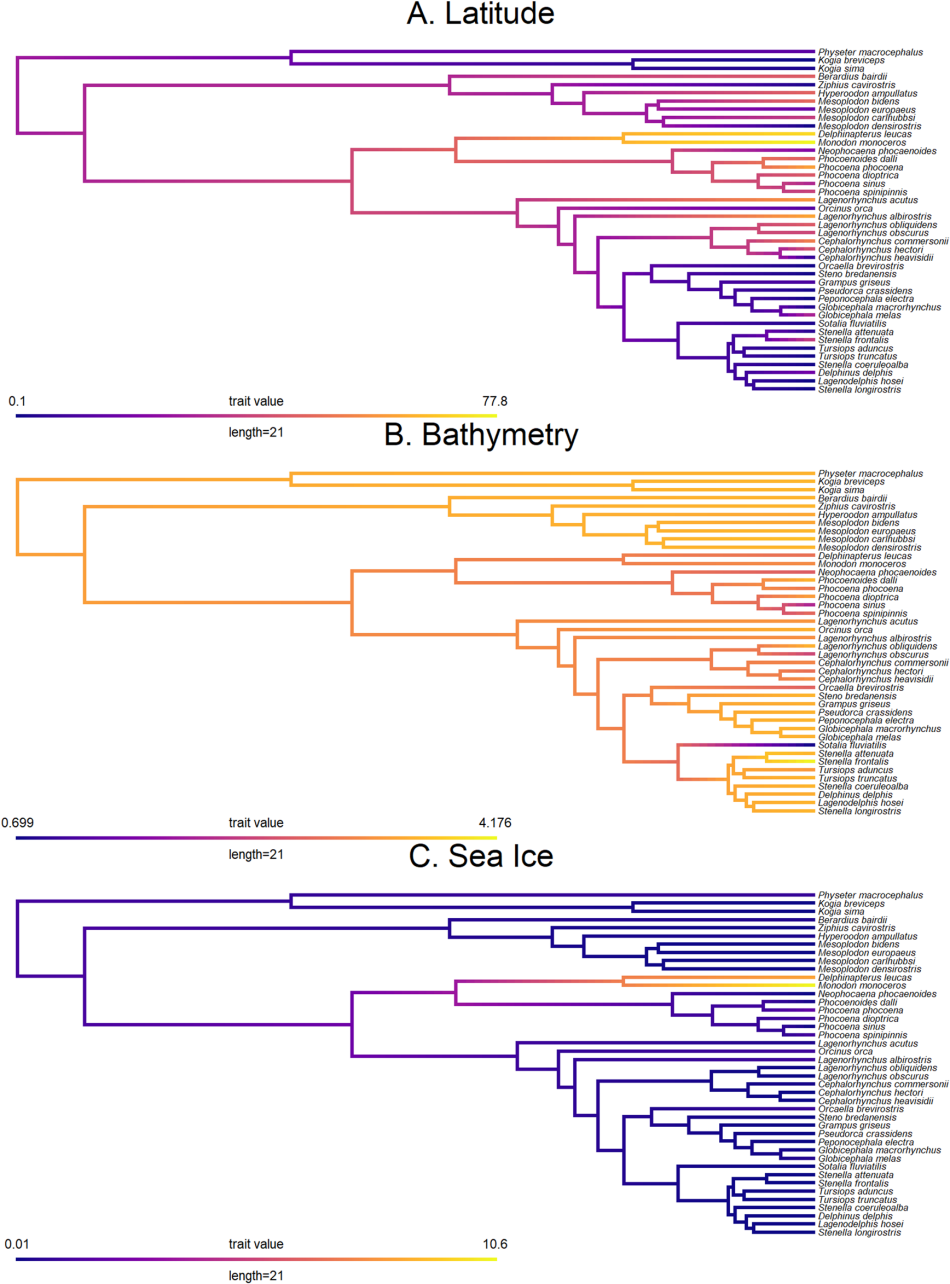
Ancestral state reconstruction of three environmental habitat variables across a phylogeny of 42 odontocete species. Phylogenetic trees show the evolutionary history of: (**A**) absolute latitude, (**B**) bathymetry (mean habitat depth), and (**C**) sea ice season length. Trait reconstructions were performed using maximum likelihood estimation under a Brownian motion model of evolution. Color gradients along branches represent inferred ancestral states, with yellow to purple shading corresponding to low-to-high trait values (see horizontal color scale bars). Specifically, stronger purple hues indicate higher absolute latitude (**A**), deeper habitats (**B**), and greater sea ice season length (**C**). These visualizations highlight multiple transitions in environmental habitat use over evolutionary time, revealing that some lineages have independently shifted into colder, deeper, or more ice-associated habitats

We contrasted phylogenetic relationships among the odontocete whale species based on the three environmental variables (latitude, bathymetry, and sea ice) (Fig. 6). Environmental selection has evolved across odontocete cetaceans with early ancestors appearing to have occupied mid-latitude habitats, with multiple lineages (e.g., genera *Delphinapterus*, *Monodon*, and *Mesoplodon*) showing transitions toward high-latitude environments. Conversely, some taxa (*Orcinus*, *Tursiops*) have retained or shifted toward lower-latitude distributions. Deeper-water preferences appear to have evolved independently in multiple lineages, including in the common ancestor of Kogiidae and Physeteridae, within Ziphiidae (Slow whales), and in some members of Delphinidae. In contrast, taxa frequently associated with continental shelves, such as *Delphinus* and *Tursiops*, generally exhibit shallower habitat use, although habitat preferences within these genera vary across their geographic ranges. Ancestral odontocetes likely did not inhabit sea ice-dominated environments, but Arctic-adapted species (*Delphinapterus*, *Monodon*) show independent evolution of seasonal ice tolerance, reflecting ecological specialization, while other species such as *Phocoena Phocoena*,* Physeter macrocephalus*,* and Orcinus* orca occur in seasonally ice-covered habitats in parts of their range, indicating some degree of ecological flexibility that includes ice-associated environments. Together, these reconstructions suggest that odontocetes have repeatedly adapted to polar and ice-associated environments across multiple lineages, while deepwater specialization appears to have arisen within specific clades, such as a single evolutionary origin in Ziphiidae.

## Discussion

Our conclusions, based on broad-scale patterns that are robust to expected intraspecific variability, identified three distinct life-history strategies among 42 odontocete species, which we term Bet-Hedging, Fast, and Slow whale LHS (Table 4). These groupings reflect divergent evolutionary responses to ecological pressures and life-history trade-offs and support a more diverse view of life-history evolution than a simple fast-slow perspective [109]. However, many species occupy an intermediate position in life-history space which highlights that not all species align closely with defined strategy endpoints; rather, many species tend toward a given strategy while retaining mixed or context-dependent traits. It is important to note that our inferences are constrained by uneven data availability across cetacean taxa, reflecting the inherent challenges of studying long-lived marine species that spend their entire lives in the ocean and are only partially accessible to direct observation. Species in the Bet-hedging LHS exhibit a conservative strategy characterized by delayed maturity, long lifespans, and investment in relatively few, well-developed offspring—traits typically associated with high parental investment and reproductive risk-spreading in unpredictable environments. In contrast, Fast LHS whale species display life-history traits aligned with faster strategies, including earlier maturation and higher reproductive output, consistent with more opportunistic or resilient demographic profiles. The Slow LHS species tend toward being distinguished both morphologically and reproductively, possibly reflecting specialization for deep-diving in pelagic habitats, with unique life-history traits not easily aligned with classical r/K selection models [33]. For example, the more robust body shape may be an adaptation to deep diving in an open ocean environment where refugia is lacking [37, 46]. This approach enabled us to disentangle direct and indirect effects of environmental variables on life-history traits and to identify the most plausible evolutionary pathways linking ecological conditions to reproductive strategies among odontocetes.

**Table 4:** Summary of Odontocete life-history traits by life-history strategies (LHS)

LHS	Example Taxa	Life-History Traits	Ecological Context
Bet-Hedging	Monodontidae (e.g., Narwhal, Beluga)	Late maturity, small offspring, low parental investment	High-latitude, seasonal sea ice
Fast	Delphinidae (e.g., Bottlenose dolphin,)	Early maturity, frequent reproduction, short lifespan	Coastal to mid-latitude environments
Slow	Ziphiidae (e.g.,, Blainville’s Beaked whale)	Unique morphology, slow reproduction, extreme deep-diving ability	Deep offshore habitats

Clustering of species within these LHS generally follows taxonomic patterns, suggesting that both deep phylogenetic constraints and ecological adaptations have shaped life-history evolution within odontocetes. Notably, the placement of the Arctic-adapted Monodontidae (i.e., narwhal and beluga) within the Bet-Hedging LHS indicates the role of high-latitude seasonality and sea ice dynamics in selecting for decreased offspring investment and long life [82]. Beluga and narwhal exhibit remarkably similar ecological and life-history patterns, reflecting their shared evolutionary history and adaptation to high-latitude, ice-associated environments [27, 76]. Both species occupy Arctic regions characterized by seasonal sea ice, relying on dynamic ice edges and polynyas for access to prey and respiration. Their life histories are marked by slow growth and extended maternal care—traits consistent with survival in extreme and seasonally variable habitats [68].

A similar pattern is also evident in killer whales, although their inclusion within or near the Bet-Hedging strategy should be interpreted cautiously given substantial ecotypic variation in habitat use and diet. In particular, resident and transient ecotypes in the North Pacific differ in their reliance on high-latitude environments and exposure to seasonal prey availability [86].

Phylogenetic reconstruction and trait mapping describe how distinct life-history strategies are distributed across evolutionary lineages, with some convergence observed among unrelated odontocete species. While the Bet-hedgers exhibited the greatest mean range latitude around 25°, latitude overall did not statistically differentiate the three LHS, possibly reflecting high ecological variability. For example, killer whales are considered Bet-hedgers and have a global distribution with greater densities at mid-latitudes and genetic evidence of diversification at high latitudes [45, 84]. This lack of differentiation may partly reflect the broad, often transhemispheric distributions of many whale species, whose ranges span both northern and southern hemispheres and encompass wide latitudinal gradients. Such extensive geographic ranges likely reduce detectable latitudinal contrasts among life-history strategies, thereby obscuring potential ecological or evolutionary differences associated with latitude. Similarly, although Slow and Fast whale species were more associated with habitats with less sea ice compared to Bet-hedgers, these differences were not statistically robust. In fact, the use of sea-ice areas is somewhat common for some Slow whales [24, 35] which is also confounded by the number of species that have ranges in both hemispheres resulting in an overall average latitude of close to 0^o^ (e.g., sperm whales). The evolutionary rates of genes associated with deep-diving have been documented as notably higher in Slow whales than in other cetacean species, implying unique selective pressures in their respiratory systems and thereby facilitating diving and minimizing the risk of decompression sickness [57].

Our results highlight significant conservation implications by revealing how evolutionary history and ecological context shape odontocete life-history strategies. Although life-history traits in odontocetes often exhibit strong correlations with environmental gradients such as latitude and the presence of sea ice, these relationships weakened when controlling for phylogeny. This suggests that the observed trait-environment correlations are, at least in part, a result of shared ancestry rather than independent evolutionary responses to environmental conditions [105]. In contrast, species that occupy high-latitude and sea ice-associated habitats often belonged to a few unrelated clades, indicating a phylogenetically evolved life-history strategy suited to polar environments [38, 94]. Specifically, the evolution of traits adapted to high-latitude environments characterized by seasonal sea ice appears to have strong phylogenetic underpinnings [19]. This suggests that certain cold-adapted species—such as narwhals, belugas, and high-latitude populations of killer whales and sperm whales—possess life-history traits that are not only shaped by current ecological conditions, but also reflect deep evolutionary lineages [6, 8, 104, 115, 120]. This pattern suggests that the capacity to exploit cold-water environments may have evolved multiple times in odontocete lineages and been retained through evolutionary time, even among taxa with broad latitudinal ranges or seasonal use of high-latitude habitats [25, 47]. These species likely exhibit unique genetic adaptations to seasonal environments, including slow maturation, long lifespans, and reduced investment in offspring. As such, they may be less able to shift their ecological niches in response to rapid environmental change, thereby needing specialized conservation strategies that recognize their evolutionary distinctiveness and limited adaptive flexibility [36, 71].

In contrast to latitude and sea ice, relationships between life-history traits and bathymetry remained strong even after phylogenetic correction, indicating a different evolutionary dynamic. The relatively weak influence of phylogeny on the association with water depth suggests that deep-water specialization has evolved independently in multiple odontocete lineages [111], likely in response to ecological opportunities or constraints such as prey availability, competition, and predation pressure [54, 57, 131]. Examples include the independent evolution of deep-diving adaptations in species as phylogenetically distinct as beaked whales (Ziphiidae), sperm whales (Physeteridae), and some oceanic dolphins (Delphinidae) [83]. These convergent adaptations support the interpretation that deep-water foraging represents an ecologically driven life-history strategy [129], shaped more by habitat use and foraging ecology than by shared ancestry. An alternative hypothesis is that deep-diving capabilities may represent an ancestral trait within Odontoceti that was subsequently lost in multiple lineages, including most Delphinidae, rather than having evolved independently in distantly related groups. However, current comparative and phylogenetic evidence generally supports multiple independent origins of extreme deep-diving specializations, particularly in Ziphiidae and Physeteridae. Together, these findings suggest that the evolutionary pathways leading to life-history diversification in odontocetes differ depending on the environmental axis considered. While traits associated with latitude and sea ice season length may reflect ancient lineage-specific adaptations [14], those linked to bathymetric niche appear to have evolved repeatedly in response to similar ecological pressures across distantly related taxa [17]. Despite their independent origins, these deep-diving species tend to exhibit slow life-history strategies, characterized by low reproductive rates, long interbirth intervals, and high parental investment; however, these patterns are less well resolved in Ziphiidae due to limited data, and species such as northern bottlenose whale show intermediate or mixed life-history characteristics [42, 85]. These traits make them inherently vulnerable to anthropogenic stressors, including noise pollution, ship strikes, deep-sea resource extraction, and climate-driven prey shifts [43, 77]. The convergent evolution of these traits in disparate lineages points to common ecological constraints that limit the capacity of deep-water species to recover from population declines [61].

High-latitude odontocetes that tend toward pelagic deep-diving behavior also share slow life-history strategies, delayed maturity, low reproductive rates, and long lifespans, that limit their capacity for rapid demographic recovery [5, 16, 106]. In contrast, some mid-latitude, nearshore species such as common bottlenose dolphins (*Tursiops truncatus*) have relatively higher reproduction, greater ecological generalism, and more frequent calving [11]. These fast life-history traits may provide greater resilience to human disturbance, enabling quicker rebounds following environmental or anthropogenic stress [95].

Together, these findings emphasize that conservation planning for odontocetes must account for both ecological specialization and evolutionary history [59]. Cold-adapted bet-hedgers, such as narwhals and belugas, may require protection of ice-associated habitats, careful strict regulation of industrial activity, and long-term population monitoring, as recovery following disturbance is likely to be slow and uncertain [58]. Deep-diving Slow specialists, including *Ziphiid* and *Kogia* whales, combine slow reproductive rates with high sensitivity to anthropogenic noise, naval sonar, and deep-sea industrial activity ([43]; Wensveen 2019), and are additionally difficult to monitor because of their offshore distributions and cryptic behavior. These vulnerabilities make precautionary management of threats especially important. By contrast, mid-latitude Fast LHS species, such as delphinids and porpoises, may be less impacted due to faster reproductive rates, but still require management of fisheries interactions, bycatch, and prey depletion. Finally, preserving evolutionary diversity is crucial for maintaining the functional integrity and adaptive potential of marine ecosystems under accelerating global change [53, 63, 100].

While this study provides new insight into the environmental correlates of odontocete life-history evolution, several limitations should be acknowledged. First, precise life-history data for many long-lived, wide-ranging marine mammals are inherently difficult to obtain due to logistical, ethical, and conservation constraints [60, 70]. As a result, trait estimates may be derived from small sample sizes or captive individuals, which could influence the accuracy of our comparative analyses [31]. Although our analysis of 42 species is a relatively large dataset for cetacean biology, more species would undoubtedly improve the statistical power and robustness of our findings, particularly in underrepresented clades or ecological niches. Some understudied species have associated uncertainty or data sparse life history, and therefore there is a potential implication for the analyses and additional research is warranted. For example, *Mesoplodon bidens* (Sowerby’s beaked whale) was the only representative of the genus *Mesoplodon* (> 15 species) included in the phylogenetic analysis. This may introduce uncertainty, as *Mesoplodon* species exhibit substantial interspecific variation in body size, ecology, and life-history traits, and *M. bidens* may not be fully representative of the genus [4]. We therefore interpret results involving this lineage with caution and consider this an important priority for future analyses as additional species-level data become available. Life-history trait estimates remain uncertain and may be sensitive to methodological limitations, bias in sample procurement (e.g., strandings), and environmental variability. As an example of the latter, age at sexual maturity is known to shift in response to ecological conditions, as illustrated by changes in age at first reproduction reported for humpback whale (*Megaptera novaeangliae*; [29]), suggesting that both measurement uncertainty and phenotypic plasticity may contribute to the lack of detectable differences among groups. Advances in epigenetic ageing approaches may improve the accuracy and comparability of these estimates in the future. Additionally, the IUCN geographic range maps reflect coarse scale contemporary distributions, which may not accurately capture the historical biogeography of species and instead might mask the environmental contexts under which certain life-history traits originally evolved. The use of single representative values for environmental variables (e.g., latitude and bathymetry) necessarily simplifies the often broad and heterogeneous ranges occupied by many cetaceans; for wide-ranging species such as killer whales with the largest latitude standard deviation (SD = 40), this may obscure important environmental extremes, and thus our results should be interpreted as reflecting dominant, rather than full, environmental conditions experienced by each species. In addition, range contractions or shifts due to human exploitation and other anthropogenic impacts may have decoupled some species from their ancestral ecological conditions [26, 103, 121, 122]. Finally, there may be unmeasured ecological and evolutionary factors influencing life-history variation in odontocetes. Although life-history differences among the three LHS were detectable, their small magnitude and weak correlations with environmental variables suggest that the drivers of odontocete life-history variation are more complex and context-dependent. Our conclusions about causal links between environmental factors and life-history evolution should be interpreted with caution, as the observed relationships likely capture only part of a broader suite of interacting processes. As unmeasured factors are more likely to add noise than generate false positives [50], the true strength of some weaker correlations may be underestimated (Fig. Supplementary Material 2). Thus, our findings likely represent conservative estimates of environmental effects, underscoring the need for more detailed datasets and a paleoecological context in future comparative studies.

Future research could examine other ecological and environmental factors that likely shape odontocete life-history traits, including prey distribution and abundance, interspecific competition, and predation risk. While species-level data on these variables remain limited [20], integrating such factors with reconstructions of past environmental conditions, such as fluctuations in ocean productivity or the presence of now-extinct apex predators (e.g., *Carcharocles megalodon*), would help distinguish long-term evolutionary adaptations from more recent responses to anthropogenic change [13, 28, 87]. Linking life-history strategies to demographic and population viability data would further clarify species’ resilience, supporting conservation risk assessments and management strategies tailored to distinct odontocete groups [11]. Future work would benefit from standardized reporting of life-history trait uncertainty (e.g., confidence intervals, sample sizes, and sex- or region-specific estimates), which would enable more rigorous comparative analyses that explicitly incorporate intraspecific variability.

## Conclusions

Our findings reveal three distinct life-history strategies among odontocete cetaceans, challenging the conventional fast–slow continuum often used to describe mammalian life-history evolution ([39, 88]; V [114]). Instead, these groupings reflect a more nuanced interplay between phylogenetic constraints and ecological adaptation. In some cases, we observed convergence in life-history traits among distantly related species occupying similar habitats, highlighting the role of environmental pressures in shaping evolutionary outcomes independently of ancestry. These results underscore the need for expanded datasets and additional comparative methods to test the robustness of these patterns and uncover potentially overlooked ecological or evolutionary relationships. The conservation implications are significant for high-latitude species whose specialized adaptations make them vulnerable to rapid environmental change, and for mid-latitude and coastal odontocetes that face intense anthropogenic pressures. As large-bodied, slow-reproducing mammals, odontocetes require targeted conservation strategies that account not only for biological recovery potential but also for the preservation of social structures and cultural knowledge characteristic of long-lived individuals within populations [117, 123].

## Supplementary Information

Below is the link to the electronic supplementary material.

Supplementary Material 1

Supplementary Material 2

## Data Availability

Repository Link: https://github.com/ferguste/odontocete-lifehistory.
