# Supplementary material for: How toothed whales divide up the world: phylogeny and ecology shape life-history strategies of odontocetes
Source: BMC Ecol Evol. 2026 May 21;26:52. doi: 10.1186/s12862-026-02530-y (PMC13244838; doi:10.1186/s12862-026-02530-y)
Supplement: Supplementary file 2 — Supplementary Material 2 [file 12862_2026_2530_MOESM2_ESM.docx]

Supplementary Information


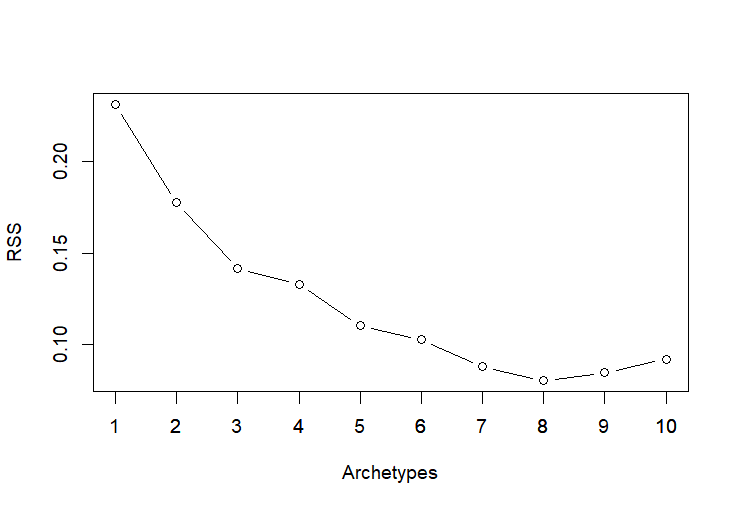


Supplementary Material 1. Relationship between the number of LHS and residual sum of squares (RSS) for 42 odontocete species based on six life-history traits and three environmental variables. The curve shows a marked decrease in RSS up to three LHS, after which improvements are smaller, indicating three groups as the optimal clustering solution.


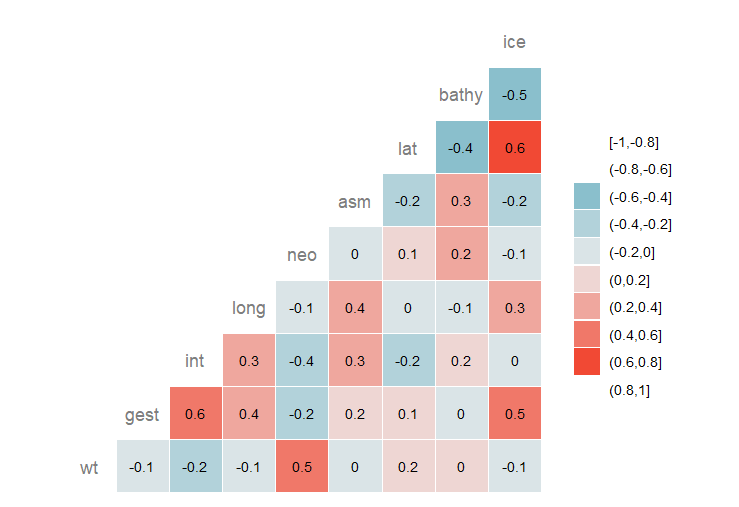


Supplementary Material 2. Statistical correlations between six life-history traits (wt = adult body mass (g); gest = gestation length (y); int = interbirth interval (mo); long = longevity (y); neo = neonate body length (m); asm = age at sexual maturity (y)) and three environmental traits (ice = mean number of months with sea ice; bathy = bathymetry; lat = absolute latitude) associated with 42 odontocete whale species. Colour code and value indicates strength of correlation coefficient.


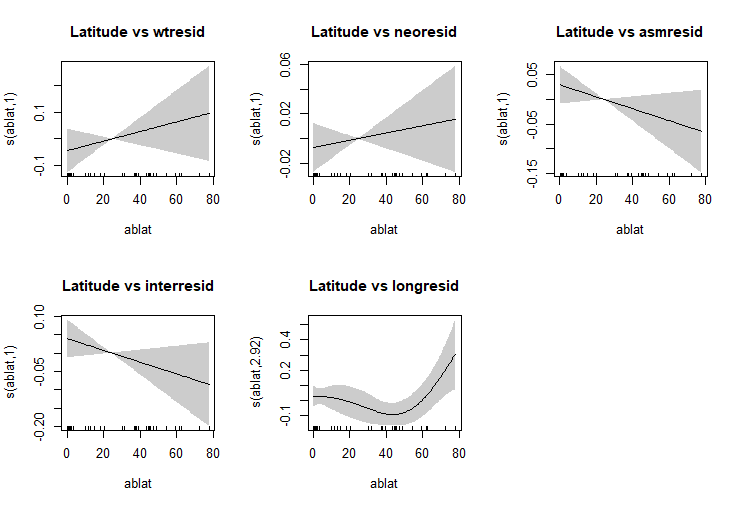

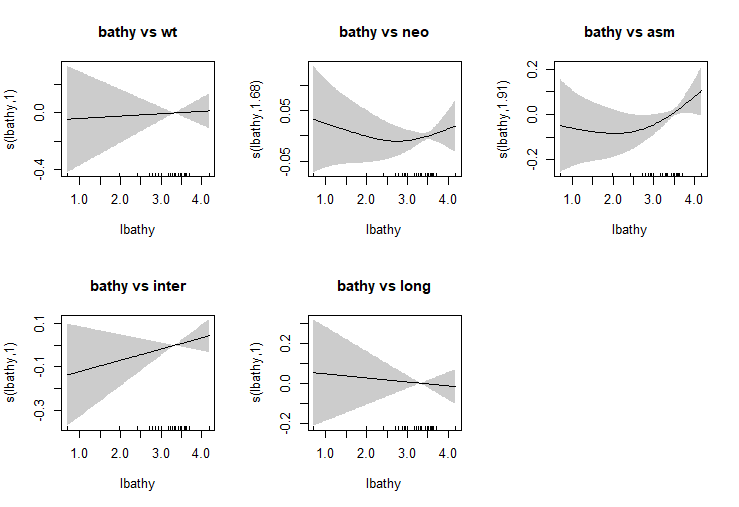

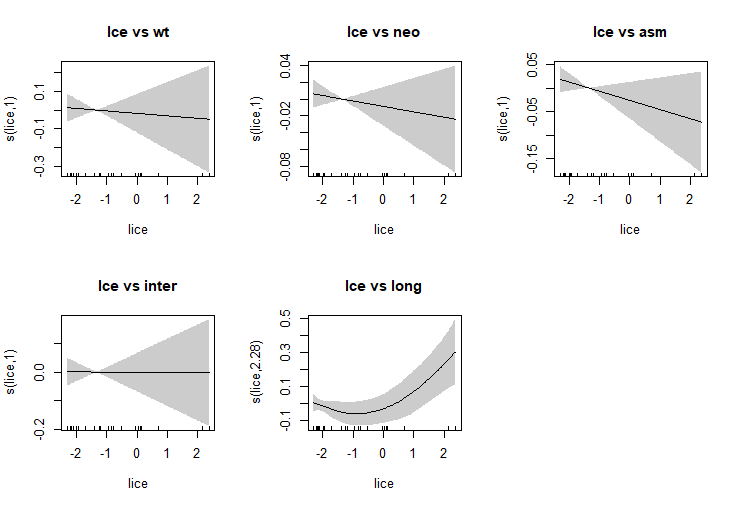


A

B

C

Supplementary Material 3. Generalized additive model (GAM) smooths showing relationships between (A) absolute latitude (ablat); (B) log-transformed bathymetry (lbathy); and log-transformed sea ice (lice) (C) and five odontocete life-history traits controlling for body mass (residuals of log-log plot on adult body length): morphology (wtresid), neonatal body length (neoresid), age at sexual maturity (asmresid), interbirth interval (interresid), and longevity (longresid). Solid lines represent fitted smooth functions, shaded areas indicate 95% confidence intervals, and rug plots show species data points.
